# Current learning strategies in fire evacuation for seniors and people with disabilities in private seniors’ residences and long-term care homes: a scoping review

**DOI:** 10.3389/fresc.2024.1305180

**Published:** 2024-02-21

**Authors:** William Thériault, Guillaume Blanchet, Claude Vincent, Isabelle Feillou, Jean Ruel, Ernesto Morales

**Affiliations:** ^1^ Center for Interdisciplinary Research in Rehabilitation and Social Integration (CIRRIS), Québec, QC, Canada; ^2^ Department of Rehabilitation, Faculty of Medicine, Laval University, Québec, QC, Canada; ^3^ Department of Mechanical Engineering, Faculty of Science and Engineering, Laval University, Québec, QC, Canada; ^4^ Department of Industrial Relations, Faculty of Social Sciences, Laval University, Québec, QC, Canada

**Keywords:** fire, evacuation, seniors, private seniors’ residences, long-term care home

## Abstract

Current strategies for teaching evacuation methods in private seniors’ residences (PSR) and long-term care (LTCH) homes may pose risks to people with disabilities (PWD) and seniors' physical and psychological health. This study aimed to address the following questions: (1) Which are the current fire evacuation learning strategies used with PWD or seniors? (2) What are the barriers and facilitators for PWD and seniors' during fire evacuation and learning strategies in PSR and LTCH? (3) What is the existing equipment that could be used with PWD seniors?. A scoping review of grey and scientific literature was done in six databases and Google scholar. Additional information was found on Québec government websites. This review identified 13 scientific papers and 22 documents. Twenty barriers (personal = 9, environmental = 11), and 14 facilitators (personal = 4, environmental = 10) were extracted. The current fire evacuation learning strategies currently used can be grouped into three categories: drills; training; promotion of a fire safety plan. Six types of evacuation equipment were found; however, their use has been scarcely documented. Safety for seniors during fire evacuation is still an important issue to be improved. Increasing awareness and creating new practices and tools that consider the strengths and difficulties of seniors seems to be a promising avenue for improving evacuation.

## 1 Introduction

Fortunately, most people will never be affected by residential fires, however for those who are, these fires can be devastating and even deadly. Residential fires are even more dangerous to human life when vulnerability becomes a common characteristic among people living in the building, such as seniors, i.e., adults over the age of 65. Indeed, in Canada, between 2014 and 2017, 40% of people who died in a residential fire were seniors and they were 2.5× more at risk than adults (1). In the United States, a 2017 study mentions that 32% of the dead during residential fires were seniors, while they represent only 13% of the population (2). Few statistics are available in Europe on the number of fire-related deaths, but the European Fire Safety Alliance (2018) (3) mentions that the seniors are among the most vulnerable, along with people with disabilities (PWD). Private seniors' residences (PSR) and long-term care home (LTCH) are very often occupied by people who have difficulty circulating and going down the stairs. Moreover, even within the normal decline in cognition and loss of some senses linked to aging, learning the right things to do in the event of a fire, as well as the evacuation itself, becomes difficult very quickly (4).

On 23 January 2014, at Isle-Verte, Québec, Canada, a seniors' residence burned to the ground, killing 32 people (5). This tragedy shows that seniors are difficult to evacuate during a fire and has also led to new regulations in Québec, such as the mandatory addition of sprinklers. However, additional measures should be put in place to ensure the safety of this population. Indeed, according to Yves Desjardins, president of the Regroupement québécois des résidences privées pour aînés (RQRA) and Cyrille Delâge, coroner on the Isle-Verte fire investigation, the addition of sprinklers alone would not be enough to ensure the safety of residents (6). Optimising the execution of a “structured evacuation”, i.e., the individual evacuation of residents in a structured and safe manner, would be the best method to preserve lives (6, 7), but the learning strategies currently used by firefighters is mainly based on evacuation drills (4, 8–11). In 2015, a survey conducted by the RQRA reported that injuries are frequent during these drills and therefore recommended the creation of alternative learning methods (12). Moreover, the drills procedure currently used in PSR and LTCH are not necessarily adapted to their capacity and living environment (4, 11), making it necessary to improve those protocols.

Thus, it is necessary to review what is currently being done in terms of learning strategies for seniors elsewhere, in order to better identify the practices to put in place. In addition, highlighting the barriers and facilitators encountered during fire evacuation and while learning fire evacuation protocols will help to create new learning strategies that consider the disabilities, strength and the realities of seniors and their caregivers. Therefore: (1) Which are the current fire evacuation learning strategies used with PWD or seniors? (2) What are the barriers and facilitators for PWD and seniors' during fire evacuation and learning strategies in PSR and LTCH? (3) What is the existing equipment that could be used with PWD or seniors?

## 2 Methods

Two scoping reviews have been realised, following the steps of Arksey and O'Malley (13), one with the scientific literature and one with the grey literature using the Prisma guideline (14). A scoping study is justified here since it aims to map rapidly the key concepts underpinning a research area and the main sources and types of evidence available, especially where an area is complex or has not been reviewed comprehensively before (15). Furthermore, it is necessary here to search the grey literature, as the subject of this study have a practical perspective.

*Find the relevant studies, through the usual means*. The scientific scoping review was realised the 26th of June 2020 to assess the current practices in terms of evacuation of seniors in case of fire in private seniors' residences and long-term care home. Supplementary Table S1, shows the four different databases used for this research: PubMed, CINAHL, PsycNET and Web of Science. The basic keywords used in those databases were separated in four concepts (Seniors, Location of residence, Fire, Evacuation) and additional terms have been used in PubMed, CINAHL and PsycNET, according to their respective thesaurus, to improve the research. Additional articles found in the bibliography have also been considered. The scoping review with grey literature was realised using 4 different ways as presented in Table 1. To begin with, some information was given by experts and partners who work in the field of fire evacuation for seniors. Then, a research using the advanced search of Google Scholar was done the 21st of September 2021 in English and in French. The keywords were based on the terms used in the previous scientific review and identified by trial and error. For the English research, the search equation used was “evacuation fire senior residence” while in French, it was its translation “*incendie évacuation aînés residence*”. Using the principle of data saturation, the first 5 pages of Google, with ten documents each, in the two languages were kept. Furthermore, specific searches in Québec's and Canada's governmental websites were done using Google (better search engine then most governmental websites). More specifically, a search was also done in the Integrated Health and Social Services Center of Chaudière-Appalaches (CA) website. Indeed, the region of CA in the province of Québec, Canada, is currently implementing a number of experimental measures for the evacuation of seniors. Finally, a specific search was conducted on Orbit, EspaceNet and Google Scholar to generate ideas for concepts that are currently used or could be used to evacuate seniors or PWD making them more difficult to evacuate. The research equations were refined using the same process as for the main research question.

**Table 1:** Data sources, date and research equation for grey literature related to evacuation in case of fire in private seniors’ residences and long-term care home—English and French.

Source	Date of search	Research equation
Specific search in google	20 September 2021	- Évacuation aînés site:cisssca.com - Gouvernement du Québec incendie aînés - Évacuation aînés site: Québec.ca - Gouvernement du Canada incendie aînés - Évacuation aînés site:canada.ca
Experts and partners		- NA
Google scholar		- Evacuation fire senior residence - Incendie évacuation aînés residence
	14 September 2020	- (Dispositif OR Device OR Gadget OR Apparatus OR Equipment) AND (Évacuation OR Evacuation OR Movement OR Transportation OR Carrying) AND (“Personnes âgées” OR Aînés OR Elders OR Senior OR Bedridden OR Disabilities) AND (Urgence OR Emergency OR Crisis OR Danger* OR Fire) - (Device OR Gadget OR Apparatus) AND (Evacuation OR Transportation OR Carrying) AND (Elders OR Senior OR Bedridden OR Disabilities) AND (Emergency OR Crisis OR Danger* OR Fire)
Orbit		- (Dispositif OR Device OR Gadget OR Apparatus OR Equipment) AND (Évacuation OR Evacuation OR Movement OR Transportation OR Carrying) AND (“Personnes âgées” OR Aînés OR Elders OR Senior OR Bedridden OR Disabilities) AND (Urgence OR Emergency OR Crisis OR Danger* OR Fire)
EspaceNet		- (Device OR Apparatus) AND (Evacuation OR Transportation OR Carrying) AND (Elders OR Senior OR Bedridden OR Disabilities) AND (Emergency)

**Figure 1:**
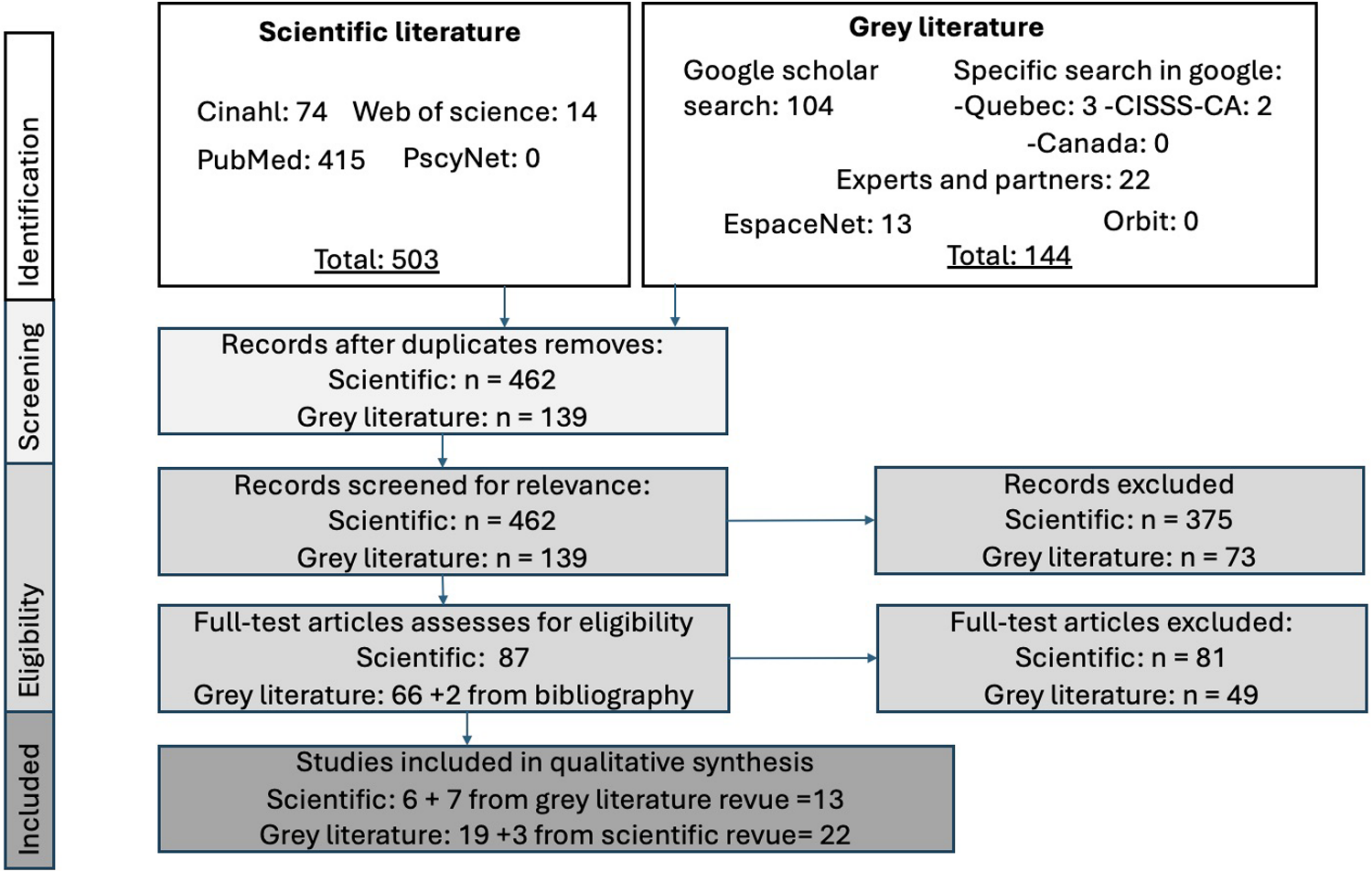
PRISMA flow chart illustrating the different phases of the selection process of relevant studies on fire evacuation in private seniors’ residences and long-term care home for the PWD and seniors and the associated numbers.

*Select the studies that are relevant to the question(s)*. For both scoping reviews, to ensure a solid internal validity, the articles were sorted out independently by two of the co-authors using the inclusion and exclusion criteria presented in Table 2 and according to the Prisma guideline (14). Figure 1 shows the steps of PRISMA. For the scientific revue, 503 articles were found, but 41 where duplicate. Of the remaining 462, a first selection was made by analysing the title and abstract to keep 87 of them. A second selection was done after reading the full text and reduced the number of articles to six. However, three articles found in the scientific revue where add to the grey literature revue. For the grey literature, 144 articles were found with five duplicates. A first selection was done by looking at the title and an overview of the text. Of the 139, 66 were kept and two more articles were added from bibliography. The second selection was done by analysing the documents using the five criteria of the AACODS checklist (Authority, Accuracy, Coverage, Objectivity, Date and Significance) (16). In this checklist, all articles that had a “No” on the significance category, were rejected, as well as articles that had more than two “No” in the other categories. For the articles that had only one, it was looked at carefully to decide if it was accepted or not. Of the 66 documents to be rated, 19 were included in the grey literature revue and seven where add to the scientific review. Thus, in total, 13 articles were kept for the scientific review and 22 for the grey literature review. Throughout the process, if any disagreements between the two readers occurred, a consensus was reached following a discussion between the two. If necessary, a third co-author was brought in to settle the issue.

**Table 2:** Inclusion and exclusion criteria for scientific and grey literature.

Inclusion criteria	Exclusion criteria
1. Mainly private seniors’ residence and long-term care home or equivalent setting. Other health care settings are accepted	1. Evacuation takes place over several days or they had several days’ notice (evacuation is not rushed) (e.g., hurricane, forest fire approaching…)
2. Addresses evacuation of seniors or people with mobility impairments or with cognitive issues requiring care	2. Article written in a language other than French or English
3. Addresses protocols, guidelines, etc. for evacuation	
4. Discusses alternative evacuation procedures or tools	
5. Discusses statistics related to evacuation	

*Chart the data, i.e., the information on and from the relevant studie*s. Data extraction was done by two co-authors and transcribed in Supplementary Table S4 (scientific literature), 5 (grey literature) and 6 (equipment) and can be found in the Supplementary Material. The first papers were looked at by each co-author and compared together to ensure the same understanding of the table and the important information to gather. The results column in the Supplementary Table S4 and S5 was created using the Person-Environment-Occupation model (17), since it is a model commonly used by occupational therapists, of whom two authors are. For the purpose of this paper, the term occupation is referred as the action of practicing/learning evacuation techniques as well as performing the evacuation itself. It allows for a focus on the individual by highlighting the interactions between these three concepts and thus makes it easier to identify the barriers and facilitators present during an evacuation and during the application of the learning strategies. In the case of patents, a description of those and of their utility was add in Supplementary Table S6.

## 3 Results

*Collate, summarize and report the results*. For scientific results in Supplementary Table S4, 13 studies are reported. All research designs are exploratory, since there were three surveys, two case studies, one observational study, three qualitative research, one retrospective study report, one pre-post study design and one repeated measure randomized block experimental design. Samples varied between nine and 416. For results in Supplementary Table S5, the grey literature where mostly guides (four government guides and one evidence-informed recommendations summary), documents describing the situation of seniors in case of firing (one review of the situation, one final research report, one doctoral dissertation, one summary of government statistics) and one policy perspective. For results in Supplementary Table S6, the 12 documents are patents. Results will be summarized regarding the three research questions.

### 3.1 Existing fire evacuation learning strategies used with PWD and seniors

There is currently a gap between perceived and real preparedness as shown by the difference between the disaster plans and the result of the drills, specifically, in the case of dementia-specific awareness (18). Despite this fact, several methods are used to limit the impact of a fire, both in terms of prevention and to facilitate evacuation.

#### 3.1.1 Drills

Evacuation drills are currently used but are also a debated method. Although it seems to help seniors to learn evacuation methods and safety tricks, few of them participate in it (18, 19). Moreover, there are risks to their physical and psychological health due to the stress involved in practising evacuation (18, 20). On the other hand, verbal or written instructions alone are also not enough, hence the importance of drills (21). To be effective, these exercises should be done at least once or twice a year (22, 23). At some of those, it was noted that there were increased numbers of people being advised by fire marshals during the practice and it was often rare that residents were evacuated to the end or to the nearest safe area. Indeed, they were often all brought to the same place, even if another area was closer (24). Another method of carrying out these practices is to include only staff. This helps to educate them on how to respond to a fire situation and how to deal with the residents (25). This type of training should be carried out minimally when there is a new employee and especially when there is a big change in personnel (23).

#### 3.1.2 Training

Training beside drills should be given to residents and care-providers (21, 25–29). It should include a diversity of resources (26) and use various senses (21) to make learning easier for them. For example, a two-day training program to train staff, including a teacher's guide, a DVD of instruction and a CD containing other useful resources seem to work to give care-providers a better understanding of the disaster plan and of the best approach in case of fire. Inclusion of specific methods on training adult learners is also beneficial (27). In terms of content, those trainings should explain the four stages of evacuation (detection of the alarm/fire, understanding and undertake the evacuation, moving, relocating) included in the safety plan to help the staff and the residents understand the particularity of each stage and the procedures to follow (28). Furthermore, those instructions should be placed on each floor of the residence (28). Indeed, easy-to-access information is a good way to spread the information (29). Care-providers should also have training on how to respond and guide person with Alzheimer's disease (29) and how to help mitigate psychological distress and anxiety (29). It is recommended for people with dementia to give them calm and proper instructions, lots of reassurance and close supervision. It is important to have a good identification of all the residents and to include specific training to deal with behavioural symptoms (18). In addition, a suggested evacuation method to teach is to first assist residents near the fire and then evacuate the other systematically (23). It is also recommended to match the evacuation speed at a level that minimises risks for seniors (30). Furthermore, education on evacuation methods should no longer be only provided by first-responding agencies, but also by other partners to improve the dissemination of information (29, 31). An example would be to use the care-providers, who are known by the residents, to teach (26).

#### 3.1.3 Promotion of a good fire safety plan

It is important to promote a good fire safety plan to help fire prevention and safe evacuation. This helps improve knowledge, role and responsibilities of everybody, good coordination of actions and promotes a rapid response at the start of the fire (30). For example, in Québec, a revision of the plan is recommended at least annually (25) while evaluating the mandatory measures in place in PSR are the responsibility of the municipalities (32).

#### 3.1.4 Research

Research should be promoted to support the development of a framework to measure the levels of preparedness of care institutions and to use the existing evidence to support planning and regulation in terms of fire prevention (29).

### 3.2 Barriers and facilitators for PWD and seniors' during fire evacuation and learning strategies in PSR and LTCH?

#### 3.2.1 Barriers for seniors' fire evacuation in private seniors' residences and long-term care home

##### 3.2.1.1 Personal factors

Several personal factors may influence the performance in an evacuation. To begin with, many seniors will hesitate before exiting at the sound of the alarm and need additional guidance or encouragement before exiting (24, 33). Thus, some of them, lose several minutes, putting their lives at risk (33). Secondly, in terms of preparation for a disaster, motivation would not be influenced by experience or taught knowledge. Indeed, the uncertainty that it will happen as well as the probability of dying beforehand demotivates seniors (26). In addition, the anxiety associated with disasters, like a fire, can be so important that some may be too afraid to think about it, and thus, do not prepare themselves accordingly (26). Many also feel that it is not their responsibility to be prepared (26). Lack of participation in fire drills and unfamiliarity with the building fire plan are personal actions that directly increase the chances of death or injury in an actual fire (19). Furthermore, a perceived safety net provides a false sense of security and prevent them from fully understanding the risk of injury or death due to fire (19). The language barrier and the wide range of educational levels can also affect the implementation of a training programme for staff (27).

Several physical limitations can make evacuation more difficult. First, people with impaired mobility may be at risk of falling, especially on stairs, given that lifts are unavailable during a fire (21, 28). Others may be unable to manipulate certain handles or doors on the escape route, particularly due to diseases such as arthritis (21, 28). Respiratory problems may also make evacuation more difficult by increasing the demand for oxygen due to the physical effort required and the level of stress. When fire and smoke are present, symptoms of respiratory dysfunction and distress are also exacerbated (21). People with hearing problems may not hear alarms or additional information given during the evacuation (19, 21, 28, 30). Those with visual problems may have difficulty orienting themselves and reading maps and signs, especially due to lack of lighting and smoke (21, 30). Furthermore, in emergency situations, bedridden or severely disabled residents may be totally dependent on the staff, leaving those with milder disabilities unattended (19).

Cognitive problems as well as dementia can also affect evacuation. Indeed, these individuals have special needs during an evacuation and are more vulnerable, due to difficulties in concentration (18, 30), comprehension (28, 30), memory (21, 30), following instructions (18) and due to behavioural problems, such as anxiety, distress, wandering or agitation (18, 21). In addition, high anxiety conditions make memory issues worse (30). Sleep inertia can also occur for seniors and reduce physical and cognitive performance for at least 30 min after awakening (21). Medications can also play an important role in the cognitive ability of seniors, including decreasing their reaction time and alertness.

##### 3.2.1.2 Environment factors

At the environmental level, it has also been found that most fires in private residences in Québec start in residential areas, mainly in the kitchen and bedroom (34). Several environmental barriers can hinder drills or the evacuation itself. First, at the institutional level, the lack of time to organise drills (27), the lack of resources in residences (35) and the shortage of staff, mainly at night (19, 24), are the three main difficulties identified. A lack of regulation, mostly in relation to people with disabilities, is also present (36). Secondly, the lack of preparation and materials is also problematic. Indeed, ineffective manuals, not having cards to indicate empty rooms and locked doors without an accessible key, are all elements that can hinder an evacuation for employees. Poor lighting at the evacuation plans can make it difficult for residents to evacuate (20). Thirdly, on an architectural level, the type of construction often used for residences common areas, for example, large rooms (cafeteria, playroom, lounge, chapel, etc.) and long, wide corridors, favour the spread of fire and smoke (19, 37). The lack of sprinklers in many residences (19, 28, 35) is also problematic, as is the absence of an alarm system linked to the fire department, preventing a rapid response from the firefighters (28). Moreover, buildings without firewalls prevent the effective implementation of horizontal evacuations, which is easier to achieve than vertical evacuations (28). Finally, clutter, grouping, lack of handrails and visibility, are all factors that can hinder an efficient and safe evacuation (28).

#### 3.2.2 Facilitators for seniors' fire evacuation in private seniors' residences and long-term care home

##### 3.2.2.1 Personal factors

In terms of personal factors, it is recommended that managers ensure that their employees and replacements have received fire safety training (22, 38) and that residents are informed about fire safety and good behaviour (22). This ensures that everyone knows their role and avoids confusion, thus limiting the danger to residents and employees (38). Good communication between staff, firefighters and the incident coordinator is also desirable to facilitate evacuation (38). In addition, in the event of a disaster, it was found that older residents are willing to follow the instructions of government and municipal response agencies (fire and police departments, military), especially among those with previous disaster experience (31). According to Proulx (33), 89% of people in a fire practice followed instructions from the voice communication system and 64% of them found the information useful. From the same study, 81% said they would like to obtain more information on fire safety.

##### 3.2.2.2 Environment factors

It was found that in most fires between 2016 and 2019 in private seniors' residences in Québec, the smoke alarms and the alarm system worked well (34). In addition, even though private seniors' residences often had a less developed security system than public ones, people felt safe because of the presence of staff (32). Several environmental facilitators can play a role in evacuation. First, a good communication system (21, 23, 38), with a clear and defined ordering hierarchy (21, 37) greatly assists the police and staff in evacuating residents. Secondly, an audible alarm system and voice communication system are effective means, especially when coupled together, to warn residents, even those with hearing problems (33). The alarm system can also include a strobe light to warn people in a deeper sleep (e.g., medication) or with hearing problems (21). In addition, the fire service should be alerted first by the alarm system rather than by employee action (23). Thirdly, safety can be greatly increased by high standards of construction (fire-resisting construction and compartmentation, incorporating sub compartments with a small number of bedrooms, fire-rated, bedroom doors with smoke seals and automatic closure on alarm). This protects residents and makes evacuation easier for employees (23, 37). Sprinklers and free-swinging automatic door closers also reduce the spread of fire and smoke (23, 37), while the presence of handrails and emergency lightning in exit door and corridor facilitate the movement of people with reduced mobility (21). Fourth, a clear evacuation plan (21, 22, 32) and maps showing a well-marked and lit path is very important to facilitate evacuation as it allows a better understanding of the path to be followed by the residents (21, 30).

### 3.3 Existing equipment or invention

Several inventions have been tested over the last decade to help evacuate in emergency situations people with or without disabilities. Twelve patents have been found and can be regroups into six types of equipment. Some of them are similar to a chair, making it easier to get down the stairs than a manual descent, promoting ideal positioning of the carer and improving comfort for the resident. This type of chair can be motorized or not (39–41). About these types of chairs, a focus group with firefighters also pointed out that they preferred devices with a longer extension at ground level in order to be able to cover three steps at a time, thus offering better stability to the device (40). There are also different types of boards to facilitate the transport of residents, some of which can be slid along the floor by a single carer (42) while others must be lifted by two people (43, 44). An apparatus can also be installed on beds to help transport bedridden people (45). Others allow residents to be lowered by means of a lift system, which can be either inside (46, 47) or outside the building (48, 49), or by using a zipline (50) or a tube on the side of the building with a padded sleeping bag (51).

## 4 Discussion

This scoping study has answered the first research question since it identified three kind of learning strategies (drills; training; and the promotion of a good fire safety plan) The second research question has highlighted nine personal barriers and 11 environmental barriers, as well as four personal facilitators and ten environmental facilitators for seniors' fire evacuation strategies in PSR and LCT homes. The last research question pointed out six types of equipment/invention through 12 patents: chairs, boards, apparatus for beds transportation, lift system, zipline and tube. Given those results, the discussion will be presented in two main parts: *prevention and learning strategies* regarding what to do in case of fire; and tools uses to help *evacuate* PWD and seniors.

### 4.1 Recommendations for prevention and learning strategies

One of the major points that emerges is it seems that there is a crying need for awareness-raising at several levels. Indeed, on an individual level, it is necessary to ensure that seniors understand the implications of not being prepared in case of fire: they are not only putting their lives at risk, but also the lives of those who will come to try to help them. At the community and institutional level, we need to ensure that residences have the resources to train their staff and residents in fire prevention and evacuation methods. Indeed, it seems there is also a need to continue to improve the learning strategies currently used. One way to do this would be to not only rely on drills, but also to add training and practices provided by the community. An example of such training could be a peer training program (52) to involve more seniors in the process and to build on social reinforcement as an awareness raising method. Considering the difficulties of the residents, especially at the cognitive level, during drills and training programs it is essential to ensure that they understand and integrate the lessons. For example, short learning segments could make it easier to keep their attention and concentration, thus maybe facilitating their understanding of the subject (30). To ensure that as many people as possible participate, it could be possible to implement these training capsules through already organised group or social activities.

Given the complexity of the problem, a multi-approach is necessary to help both residents and staff to learn. Including different senses and ways of learning would give a more complete experience and help build on each person's strengths. Evacuation drills remain an essential learning factor for residents and staff, but on the other hand, they can be harmful to the seniors, both physically and psychologically. It would therefore be interesting to develop alternatives methods of giving them practical training (53). For example, the creation of a serious games (54), using technology as a medium, could simulate a fire situation in a realistic way and offer a learning opportunity with less risk for the seniors. In addition, this type of practice could also be developed for staff. Indeed, conducting a drill is time consuming and difficult to organise at high rate of occurrence (10, 11). Although this has not been tested yet, a serious game simulating drills could be a venue to explore, by provide training for new staff and serve as a reminder to older staff, potentially reducing the need for frequent actual real drills.

Another area that needs improvement in terms of prevention is the environment. The residences must have the resources to create the safest possible environment and have the necessary equipment to evacuate quickly, efficiently and in a manner that is safe for all. It is important to ensure the best architectural layout and equipment to facilitate communication between firefighters, employees, and residents. Many alarm systems can be used to alert residents in different directions, but it is also important to ensure that these systems do not incapacitate others. High volume and strobe lights can be disruptive to some people, especially those with sensory problems such as autism spectrum disorders or intellectual disabilities (55–57). This seem relevant to be taken into consideration mainly because the longevity of these two populations has increased significantly (58, 59), and they may find themselves more often in residences. It is also important to ensure that systems to slow the spread of fire are not harmful to residents. Indeed, due to their physical and sensory difficulties, some tools can become a nuisance. For example, it is also possible that sprinklers may create steam, which could lead to poor visibility and make breathing more difficult for those with respiratory problems (6).

### 4.2 Recommendations for tools used during evacuation

In order to help in the evacuation of the seniors in the event of a fire, 6 different solutions (chairs, boards, equipment for beds transportation, lift system, zipline and tube) were found in this review. However, their use has been little documented in the literature. One possible explanation for this is the cost associated with these equipment's. Indeed, many of them may be expensive and residences cannot afford them. Thus, it would be beneficial to continue to work on the development of evacuation tools/solutions for the seniors, taking into consideration not only the cost, but also the physical difficulties, such as decreased overall strength, grip and breathing problems, and cognitive difficulties, such as anxiety, dementia and comprehension problems. These prototypes should also take into consideration the level of learning required for use, the usability and inclusiveness of the equipment for various clienteles and staff who will manipulate them, as well as the environment in which it is used, both in terms of the type of building and the weather conditions if it is outdoors. We can especially think of the impact of winter on the usability of the equipment in the case of northern countries. To ensure that theses inventions are used, partnerships could be formed with governments, community organizations along with public security and firefighters' departments to facilitate the dissemination of those evacuation methods in private seniors' residences and long-term care home. Some of these partnerships could be used to help finance the cost of the equipment, while others could be used to help raise awareness of the need for proper equipment and how to use them.

### 4.3 Limitations of the study

This scoping review only sample articles from certain literature databases and journals, along with a limited number of pages from Google Scholar, and English and French-only documents or articles, which may compromise to some extent, the external validity of the review.

## 5 Conclusion

This review helped to identify ideas of what is considered as current learning strategies when it comes to teach fire evacuation in homes for the seniors. It has also highlighted that a multitude of barriers and facilitators may affect the learning process of the protocol, but also the evacuation itself. There is still a long way to go to ensure that the learning strategies used and evacuations themselves are safe for all. Increasing awareness is a pivotal first step and increasing the amount of scientific literature on the subject will help to achieve this goal. The development of new methods that are more adapted to the experiences of seniors, such as a formation that considers their strengths and difficulties and that also focuses on their lifestyle to increase their adherence, might be a way to make it easier for seniors to learn the evacuation guidelines. In order to ensure that the formation is as effective as possible in transferring knowledge, it would be wise to include seniors in the creation of the course. Research, development, and dissemination of new evacuation tools would also be essential to enable safe and effective evacuation.

However, little information was found on the content of the training courses, what staff and residents should be taught and what specific guidelines should be followed in the event of a fire. Further research on this subject could therefore enable better dissemination of the guidelines and prevention methods to be followed and thus prevent deaths among a vulnerable and growing population.
