# Supplementary material for: Current learning strategies in fire evacuation for seniors and people with disabilities in private seniors’ residences and long-term care homes: a scoping review
Source: Front Rehabil Sci. 2024 Feb 21;5:1305180. doi: 10.3389/fresc.2024.1305180 (PMC10914951; doi:10.3389/fresc.2024.1305180)
Supplement: Supplementary file 1 [file Table1.docx]

**Supplementary material**

Table 1: Databases and research equations by source for scientific literature related to residence, elderly, and fire (26 June 2020).

| **Database** | **Research equation by concept** |
| --- | --- |
| Base equation for all databases | Elderly  Adult“ OR "Aged" OR "Aged, Hospitalized" OR “Aged person” OR “Aged, 80 and over" OR “Alcohol-Induced Disorders, Nervous System” OR “Articulation Disorders” OR "Articulation Disorders, Organic" OR “Aphasia” OR “Auditory Perceptual Disorders” OR "Behavioral and Mental Disorders" OR “CardioVascular Disorders” OR “Cognition Disorders” OR “Communication Disorders” OR "Communicative Disorders" OR “Chronic Disease” OR "Cognitive Dysfunction" OR “Chronic  Illness” OR “Cognitive Impairment” OR “Deaf- Blind Disorders” OR "Deafness" OR "Delirium, Dementia, Amnestic, Cognitive Disorders" OR "Developmental Disabilities" OR “Disabled Persons” OR “doyen“ OR "Dyssomnias" OR “Frail elderly” OR "Gait Disorders, Neurologic" OR “Hearing Disorders” OR “Hearing Loss” OR "Hearing Loss, Functional" OR “Hereditary Autoinflammatory Diseases” OR "Heredodegenerative Disorders, Nervous System" OR “Illusions (Perception)” OR “Intellectual Development Disorder” OR “Intellectual Disability” OR “Language Disorders” OR “Learning Disabilities” OR “Memory Disorders” OR “Mental Disorders” OR “Mild Cognitive Impairment” OR “Mentally Disabled Persons” OR “Nervous System Disorders” OR “Neurocognitive Disorders” OR “Neurodegenerative Diseases” OR “old person” OR “older“ OR "Organic Mental Disorders, Psychotic" OR “Perceptual Disorders” OR “Persons With Hearing Impairments” OR “Psychomotor Disorders” OR "Retirement" OR “senior“ OR “Sensation Disorders” OR "Sleep Disorders" OR “Sleep Wake Disorders” OR "Sleep Disorders, Circadian Rhythm" OR "Social Behavior Disorders" OR “Social Communication Disorder” OR "Somatosensory Disorders" OR "Vascular Hemostatic” OR “veteran“ OR “Vision Disorders”  Location of residence  Adult Day Care Center” OR "Alternative Health Facilities" OR “Center, Senior” OR “Centers for the Aged” OR “Centers, Senior” OR ”Day Care Centers” OR “Day Care Center, Adult” OR “Day Care Centers, Adult” OR “Day Care Centers” OR “Facility Design and Construction" OR "Hospital Design and Construction" OR ”Hospital  Environment” OR "Long-Term Care" OR “Home, Old Age”  OR “Homes, Old Age” OR “Housing for the Elderly” OR “Nursing Home” OR "Nursing Home Design and Construction" OR “Old Age Home” OR “Old Age Homes” OR ”Retirement Communities” OR “Retirement residence” OR "Rural Health Centers" OR “Senior Center”  Fire  “Blaze” OR “burning sinister” OR “control burning” OR “disasters“ OR "Fire Apparatus" OR "Firefighters” OR “Fire  Fighters” OR “Fire  Prevention” OR "Fire Safety" OR “firefighter” OR “Firefighters” OR “firefighting” OR "Firefighting Equipment and Supplies" OR “fires“ OR “firestorm“ OR “Flames“ OR "Natural Disasters" OR “Smoke” OR “wildfires“  Evacuation  "Architectural Accessibility" OR "Disaster Planning" OR "Emergency Responders" OR “Disaster evacuation” OR “Emergencies" OR “Emergency Management” OR “Emergency evacuation” OR “Emergency Preparedness” OR Emergency Services OR “Emergency Shelters” OR “Evacuation Shelter” OR “Evacuation Shelters” OR “Shelters, Emergency” OR “Shelters, Evacuation” |
| Additional terms for PubMed | Elderly  ("Adult"[Mesh]) OR ("Aged, 80 and over"[Mesh]) OR ("Aged"[Mesh]) OR ("Aphasia"[Mesh]) OR ("Articulation Disorders"[Mesh]) OR ("Auditory Perceptual Disorders"[Mesh]) OR ("Cardiovascular Diseases"[Mesh]) OR ("Chronic Disease"[Mesh]) OR ("Cognition Disorders"[Mesh]) OR ("Cognitive Dysfunction"[Mesh]) OR ("Communication Disorders"[Mesh]) OR ("Deafness"[Mesh]) OR "Developmental Disabilities"[Mesh] OR ("Disabled Persons"[Mesh]) OR  ("Disease"[Mesh]) OR ("Frail Elderly"[Mesh]) OR ("Hearing Disorders"[Mesh]) OR  ("Hearing Loss, Functional"[Mesh]) OR  ("Hearing Loss"[Mesh]) OR ("Hospitals, Chronic Disease"[Mesh]) OR ("Intellectual Disability"[Mesh]) OR ("Gait Disorders, Neurologic"[Mesh]) OR ("Language Disorders"[Mesh]) OR  ("Memory Disorders"[Mesh]) OR ("Mental Disorders"[Mesh]) OR  ("Mentally Disabled Persons"[Mesh]) OR ("Nervous System Diseases"[Mesh]) OR ("Neurocognitive Disorders"[Mesh]) OR ("Neurodegenerative Diseases"[Mesh]) OR  ("Neurodevelopmental Disorders"[Mesh]) OR ("Perceptual Disorders"[Mesh]) OR  ("Persons With Hearing Impairments"[Mesh]) OR  ("Psychomotor Disorders"[Mesh]) OR ("Retirement"[Mesh]) OR ("Sensation Disorders"[Mesh]) OR ("Sleep Wake Disorders"[Mesh]) OR ("Sleep Disorders, Circadian Rhythm"[Mesh]) OR  ("Social Communication Disorder"[Mesh]) OR ("Somatosensory Disorders"[Mesh]) OR  ("Vision Disorders"[Mesh])  Location of residence  ("Adult Day Care Centers"[Mesh]) OR ("Facility Design and Construction"[Mesh]) OR ("Hospital Design and Construction"[Mesh]) OR "Homes for the Aged"[Mesh] OR ("Housing for the Elderly"[Mesh]) OR ("Long-Term Care"[Mesh]) OR ("Nursing Homes"[Mesh]) OR ("Senior Centers"[Mesh])  Fire  ("Disasters"[Mesh]) OR ("Firefighters"[Mesh]) OR ("Fires"[Mesh]) OR ("Natural Disasters"[Mesh]) OR ("Smoke"[Mesh]) OR ("Wildfires"[Mesh  Evacuation  ("Architectural Accessibility"[Mesh]) OR ("Disaster Planning"[Mesh]) OR ("Emergencies"[Mesh]) OR ("Emergency Responders"[Mesh]) OR ("Emergency Shelter"[Mesh]) |
|  |  |
|  |  |
|  |  |
| additional terms for Cinahl | Elderly  (MH "Adult") OR MH "Aged, 80 and Over") OR (MH "Aged, Hospitalized") OR MH "Aged") OR  (MH "Alcohol-Induced Disorders, Nervous System") OR (MH "Aphasia") OR (MH "Articulation Disorders, Organic") OR (MH "Articulation Disorders, Organic") OR (MH "Behavioral and Mental Disorders") OR (MH "Cardiovascular Diseases") OR (MH "Chronic Disease") OR (MH "Communicative Disorders") OR  (MH "Cognition Disorders") OR (MH "Deaf-Blind Disorders") OR (MH "Delirium, Dementia, Amnestic, Cognitive Disorders") OR (MH "Developmental Disabilities") OR  (MH "Dyssomnias") OR (MH "Fatigue Syndrome, Chronic") OR (MH "Frail Elderly") OR (MH "Gait Disorders, Neurologic") OR (MH "Gait Disorders, Neurologic") OR MH "Hearing Disorders") OR (MH "Hearing Loss, Central") OR MH "Hearing Loss, Conductive") OR (MH "Hearing Loss, High-Frequency") OR  (MH "Hearing Loss, Noise-Induced") OR (MH "Hearing Loss, Partial") OR (MH "Hearing Loss, Sensorineural") OR (MH "Hereditary Autoinflammatory Diseases") OR  (MH "Heredodegenerative Disorders, Nervous System") OR   (MH "Intellectual Disability") OR (MH "Language Disorders") OR (MH "Learning Disorders") OR (MH "Memory Disorders") OR (MH "Mental Disorders, Chronic") OR (MH "Mentally Disabled Persons") OR (MH "Motor Skills Disorders") OR (MH "Motor Skills Disorders") OR (MH "Neurodegenerative Diseases") OR (MH "Psychomotor Disorders") OR  (MH "Sleep Disorders") OR (MH "Sleep Disorders, Circadian Rhythm") OR  (MH "Sleep-Wake Transition Disorders") OR (MH "Social Behavior Disorders") OR (MH "Organic Mental Disorders, Psychotic") OR (MH "Retirement") OR (MH "Sensation Disorders") OR (MH "Sleep Disorders, Circadian Rhythm") OR (MH "Social Behavior Disorders") OR (MH "Somatosensory Disorders") OR (MH "Vascular Hemostatic Disorders")  Location of residence  MH "Alternative Health Facilities") OR (MH "Facility Design and Construction") OR (MH "Home Nursing") OR (MH "Hospital Design and Construction") OR (MH "Housing for the Elderly") OR (MH "Nursing Home Design and Construction") OR (MH "Nursing Homes") OR (MH "Long Term Care") OR (MH "Rural Health Centers") OR (MH "Senior Centers")  Fire  (MH "Fires") OR (MH "Fire Safety") OR (MH "Fire Apparatus") OR (MH "Firefighting Equipment and Supplies") OR (MH "Disasters") OR (MH "Natural Disasters") OR (MH "Firefighters") OR (MH "Firefighting")  Evacuation  MH "Disaster Planning") OR (MH "Emergency Evacuation") OR (MH "Emergencies") OR (MH "Architectural Accessibility") |
| additional terms for PsycNET | Elderly  {Aphasia} OR {Articulation Disorders} OR {Cardiovascular Disorders} OR {Chronic Illness} OR {Cognitive Impairment} OR {Communication Disorders} OR {Hearing Disorders} OR {Illusions (Perception)} OR {Intellectual Development Disorder} OR {Language Disorders} OR {Learning Disabilities} OR {Memory Disorders} OR {Mental Disorders} OR {Nervous System Disorders} OR {Neurodegenerative diseases} OR {Sleep Wake Disorders} OR {Vision Disorders}  Location of residence  “{Day Care Centers} OR {Hospital Environment} OR {Nursing Homes} OR {Retirement Communities}  Fire   {Disasters} OR {Fire Fighters} OR {Fire Prevention} OR {Natural Disasters}  Evacuation  {Emergency Management} OR {Emergency Services} OR {Emergency Preparedness} |
|  |  |
|  |  |
|  |  |

Table 4. Results extracted from the scientific literature (date) according to barriers, facilitators, and current evacuation practice

| **Authors (year), Title**  **Research design (n=)** | **Barriers (b), Facilitators (f) and Current evacuation practice** |
| --- | --- |
| Amla (2017)  Les conditions de vies des aînées de minorités visibles dans les résidences privées de Montréal.  Semi-structured interview (n=13) | Personal factors   - Not discussed   Environment factors   - An investigation revealed that 1041 out of 1905 private residences for seniors (55%) are not protected by sprinklers (Chouinard in La Presse, 2015). Despite the tightening of regulations surrounding private residences, there are still doubts about the guarantee of safety and the quality of service offered to seniors in these private residences. (b) - They believe that the lack of resources is the cause of the suffering (low well-being, injury, abuse…) of seniors and therefore recommend that the government provide more resources to prevent these problems. (b)   Occupation – evacuation practices:   - Not discussed |
| Bonnan-White (2016)  Independent-Living Senior Communities in Disaster: Self-Efficacy and Trust in Responding Agencies  Survey (n=45) | Personal factors   - Results here do not suggest a relationship between self-efficacy and willingness to follow mandatory or voluntary evacuation orders. However, results of the willingness scales reveal opportunities for improvement for communication and empowerment. Whereas the sampled senior residents indicated willingness to follow orders from government and municipal responding agencies (fire and police departments, military), particularly among those with previous disaster experience. (f)   Environment factors   - Not discussed   Occupation – evacuation practices:   - Occasions for increasing trust or confidence in these sources may be increased through collaborative educational events and strategic planning at the local level. Emergency management planning in communities that include ILCs may benefit from periodic assessment of preparedness, willingness to evacuate, and other measures of trust in disaster response agencies. Focus should not only rest on official first-responding agencies (police, fire department, EMS and medical), and also include other partners in response that may be responsible for communicating evacuation information |
| Folk et al. (2020)  Emergency egress for the elderly in care home fire situations  Observation of 9 fire drills (staff member= 37, residents=56) | Personal factors   - Several residents exited their rooms independently or with prompting and then remained in the hallway outside of their rooms until they were prompted again or guided to the safe zone by a staff member. True autonomous behaviour is a very grey concept when considering elderly populations (b)   Environment factors   - The staff had a large impact on the evacuation of residents as the worst total times seemed to be most affected by low staff and high proportions of residents (b)   Occupation – evacuation practices:   - Limitations for using fire drill and movement data: - In some drills, more staff were noted to have played a role in the drill than were supposed to (Ex: closing doors to resident rooms, tagging doors and supervising residents in the safe zone). While it is understandable why these staff would be present during a drill for additional safety, it is not representative of a real fire. - Another trend that was seen during all but one of the observed fire drills was that of residents evacuating (either assisted or autonomous) to only one safe zone, regardless of their proximity to the second (typically the stairwell). While it makes sense why this would be done for ease and convenience during a simulation, given the mobility and mental state of many residents (eg, having residents wait in a hallway is easier and more practical than a stairwell), it is not representative of all the safe zones that residents would be evacuated to in an actual fire. - Often not all residents were evacuated, even during worst case scenario drills. In the observed monthly drills (drills 1-4), it was seen that very few residents were evacuated at all. In the later drills where more residents did participate, staff stand-ins generally took the place of residents who would have greater difficulty evacuating (uncooperative, reduced mobility, etc.). - The roles played by the drill coordinators and fire marshals present during the drills were also seen to impact the drills. During the worst-case scenario drills, the observing fire marshals and/or drill coordinators were observed to interact with or prompt the participating staff members. This ranged from telling staff that they should be tagging the evacuated rooms to telling staff that they did not have to evacuate residents to the safe zone |
| Gershon et al. (2017)  Psychosocial Influences on Disaster Preparedness in San Francisco Recipients of Home Care  Qualitative study of 50 elderly home  (semi-structured interview) | Personal factors   - Fatalism, helplessness, and social isolation impaired the predicted cognitive processes that drive initiation and maintenance of preparedness behaviors. (b) - Motivation to prepare was generally not influenced by prior experience or knowledge of disasters (b) - The uncertainty of when a disaster might occur was also a barrier to being prepared, as it was, such a long time away. Many thought they would probably be dead before the next disaster occurred. (b) - Many people are so anxious about disasters and their unpreparedness for them that planning was not possible, because, in the words of one participant, they were, too scared to even think about it. (b) - Participants generally did not feel personal responsibility for being prepared, rather they thought that other people and agencies should help them prepare (b)   Environment factors   - Not discussed   Occupation – evacuation practices:   - Another approach may be to take advantage of the access home care recipients already have to home care providers. The aides can be trained to help home care recipients become better prepared. The training and materials needed for this type of targeted training should be provided through the combined efforts of all stakeholders (e.g., health departments, home care agencies, etc.). Numerous resources are available to provide guidance, including training curricular for home care providers, and other materials from the Department of Homeland Security and other agencies and organizations. |
| Jaslow et al. (2005)  Fire Safety Knowledge and Practices Among Residents of an Assisted Living Facility  Survey (N=58) | Personal factors   - More than one-third of those surveyed could not clearly hear the fire alarm. (b) - Lack of participation in fire drills and unfamiliarity with the building fire plan are personal actions that directly increase the chances of death or injury in an actual fire. (b) - In emergency situations, bedridden or severely disabled residents may be totally dependent on the staff, leaving those with milder disabilities unattended. (b) - A perceived safety net provides a false sense of security to the respondents and dissuades them from appropriate recognition of the risk for injury or death due to fire. (b)   Environment factors   - Fires in group-living facilities pose greater-than-normal risks for several reasons including high resident-to-staff ratios (especially at night), bedridden residents, and older construction. (b) - The type of construction used in group-living facilities also contributes to the fire risk, since many buildings are designed with large, open, meeting places that facilitate the passage of smoke and toxic gases through several stories. Additionally, many older retirement facilities were built with limited exits, combustible finishes, and a lack of automatic sprinklers. (b)   Occupation – evacuation practices:   - The US Fire Administration states that by practicing simple fire safety tips, older adults can reduce their chances of experiencing a fire and subsequent injury or death - Result of the survey given to seniors in a residence:   - What will you do in case of a fire alarm:     - 28% would attempt to find the fire rather than call for help or evacuate     - 12% stated that they would ignore it     - 15% would call the front desk to inquire about the circumstances     - 21% did not answer the question at all   - 81% correctly answered to whom the first phone call should be placed upon detecting possible fire, how to correctly move (crawl) through a smoke-filled hallway (72%), and that they would stay inside their apartments if there was heavy smoke in the hallway (88%).   - 9% answered that they would run into a smoke-filled hallway rather than close the door and remain inside.   - 63% people indicated that they did not have a copy of the fire safety plan and 86% answered that they did not have a map of the fire escape routes.   - 54% indicated that they would not go to an assigned meeting place upon evacuating the building, even if it is referenced in their fire evacuation plan. |
| Koning et al. (2014)  Indoor fire in a nursing home: evaluation of the medical response  to a mass casualty incident based on a standardized protocol.  Retrospective study reports | Personal factors   - The majority of (key) staff members had been trained properly. They had been assigned coordinating roles and helped others who were less familiar with the procedures. (f) - Good communication between staff, firefighters, and the incident coordinator. (f)   Environment factors   - A good system of communication (f)   Occupation – evacuation practices:   - The communication could be ameliorated if all partners use the National Crisis Management System. If used correctly, this system produces an accurate and immediate overview of all aspects of the evacuation and will facilitate interdisciplinary communication between the incident site and chain partners. |
| May (2016)  Building Occupant Evacuation Response to Multiple Perceived False Fire Alarms  survey (N=295) | Personal factors   - The major implication of the study showed statistical significance (p < .05) between building occupant attitudes and nuisance fire alarms for participants who had not had a fire loss but knew someone who did. It was also statistically significant (p < .05) for those who had no fire loss and did not know anyone with a fire loss. These findings contributed to expanding the literature relating to attitudes and perceptions about fire alarms. (f)   Environment factors   - Not discussed   Occupation – evacuation practices:   - Not discussed |
| Mehta et al. (2014)  Evaluating the physical demands on firefighters using track-type stair  descent devices to evacuate mobility-limited occupants from high-rise  buildings.  Repeated measures randomized block experimental design (N=12) | Personal factors   - Not discussed   Environment factors   - Not discussed   Occupation – evacuation practices:   - Comparison of 5 tools to help with stair descent: This group of experienced firefighters had more things they liked than disliked about the narrow, 2-wheel, and standard devices. The opposite was true for the long-track and rear-facing devices. However, designers of these devices should consider lengthening their tracks so that three stair nosing’s can be bridged on staircases found outside the home. |
| O’Brien et Al. (2009)  A National Initiative to Train Long-Term Care Staff  for Disaster Response and Recovery  Pre-post study design | Personal factors   - The wide range of educational and sometimes differing linguistic backgrounds of the staff being trained provided another challenge in program implementation. (b)   Environment factors   - the barrier most frequently reported by participating LTC providers was an inability to dedicate time to train their staff. Most providers reported a struggle to schedule training time that was sometimes compounded by a lack of support from their corporate office and high staff turnover rates. (b)   Occupation – evacuation practices:   - 2-day training program for for specialist that will train staff within their facilities. Include: A Teacher’s Guide for the eight classroom instruction modules, independent study versions of the learning modules, a DVD of videotaped instruction, and a CD containing other resources useful for disaster preparedness. » - The PREPARE program has had a positive impact on disaster preparedness in LTC facilities. Post surveys suggest that after attending the 2-day train-the-trainer sessions, participants made important improvements to organizational disaster plans.   -there was an increase in LTC facility staff who reported conducting both functional and tabletop exercises”   - To respond to barriers found in the program:   - the modules were reviewed in relation to staff positions to determine what content was most relevant for which discipline   - Specific instruction on teaching adult learners was incorporated   - Additional information on teaching techniques and a greater number of interactive activities were included in the training guide as well as s supplemental materials provided, such as videotaped instruction and independent learning modules.   - select materials were translated into Spanish to provide trainees with greater access to the information. |
| Proulx (1999) Occupant response during a residential highrise fire  Case study  (Questionnaire=137) | Personal factors   - When occupants first became aware of the emergency, 30% indicated attempting to obtain more information by contacting neighbours or dialling 911 (b) - A majority of respondents (70%) did not attempt to obtain additional information, as they were waiting to receive instructions from the voice communication system (b) - When asked how long after you were alerted to the fire did you attempt to evacuate, the largest group (34%), indicated a delay of about 5 min before starting to evacuate. Many specified starting their evacuation when instructed by the voice communication system, which was probably between 6 to 10 min after the fire alarm sounded. (b) - 89% followed the instructions provided which were judged useful for 64% of the respondents. (f) - 81% said they would like to obtain more information on fire safety (f)   Environment factors   - 39% of the occupants were first made aware of the fire by the sound of the fire alarm and 70% included the fire alarm as one of many sources notifying them of the emergency. The second means by which occupants indicated they were most often alerted was by the voice communication system; 13% identified the voice communication system only while 42% indicated the voice communication system along with at least another source. Interestingly, all 10 respondents who mentioned having hearing limitations, were notified of the emergency by hearing the fire alarm. Among them, 8 said the voice communication was clear and it was the cue to start the evacuation for 6 of them. (f)   Occupation – evacuation practices:   - A fire occurred in a 25-storey highrise apartment building (Over two-thirds of the respondents were over the age of 65): 83% attempted to evacuate while 17% decided to stay in their apartments and initiate protect-in-place activities. During their evacuation, all occupants above the 5re 6oor encountered smoke conditions. Only 54% who attempted to evacuate managed to escape. The others had to return to their apartment (25%) or seek refuge (21%) in a neighbour’s apartment. |
| Purser (2014)  Fire safety and evacuation implications from behaviours and hazard development in two fatal care home incidents  Post disaster interview and full-scale fire reconstruction experiments | Personal factors   - Not discussed   Environment factors   - Adequate staff training and good lines of communication between staff members are important for the safety of both staff and residents, but successful staff intervention depends on the presence of adequate passive and or active systems to free staff to perform limited interventions in the fire enclosure and to provide them with an extended period in which to carry out progressive assisted evacuation of residents as necessary. (f) - A good level of safety can be provided using a high standard of fire-resisting construction and compartmentation, incorporating sub compartments with a small number of bedrooms and free swing, fire-rated, bedroom doors with smoke seals and automatic closure on alarm. (f) - The presence of these features then provides immediate protection for all occupants beyond the fire enclosure, removing this burden from staff so that they are not overextended and free to target the enclosure and occupants immediately affected. (f) - The additional installation of a full fast-response sprinkler system provides additional protection, particularly for any occupants of the fire enclosure and staff attending in the vicinity of the fire (f) - A common feature of past serious incidents has involved fire and smoke spread from open rooms and multiple occupant exposures within a few minutes in rooms with open doors. Staff members have failed to ensure rooms are closed during the incident while involved in other emergency response activities. The use of free-swing automatic door closers is therefore an important improvement. The relaxation of this requirement in sprinklered premises may result in some smoke exposure to occupants in open rooms beyond the fire enclosure, which could be serious in situations where sprinklers are slow or fail to operate. (f) - First notification of the fire service should be automatic on alarm rather than relying on a staff call (f)   Occupation – evacuation practices:   - The main basis of the fire safety strategy for care homes in the UK is fire-resisting construction. Buildings are divided into compartment and sub compartments with fire-resisting construction of structural elements, walls, doors, ceilings, and floors mostly to 30-min fire resistance or, in some cases, 60 min depending upon the size of the building and the dependency of the occupants - This primary protection is coupled with a detection and alarm system to provide early warning of fire and protected means of escape. This is designed to enable progressive horizontal or vertical self-evacuation of residents or evacuation assisted by staff away from affected sub compartments or out of the building over the necessary timescale and avoiding exposure to smoke or heat - The basic evacuation strategy is then to first assist residents immediately involved near the fire area to evacuate and then continue to evacuate others systematically over a prolonged period [8]. This strategy has the dual benefits that fires are mostly confined to small enclosures, with limited ventilation and smoke spread, and that only a small number of occupants should be immediately threatened. A progressive evacuation of areas immediately affected by a fire incident can be implemented by small numbers of staff, and a temporary defend-in-place strategy, or gradual evacuation over a prolonged period, can be used for other occupants. - Training should include a drill at least once a year and notices listing the procedures should be displayed in a conspicuous position in a staff room or office. Training should be given at the start of a person’s employment, whenever there is a change in fire risk and at least twice every year. The number of staff in any particular Care Home will depend on specific local circumstances, but minimum requirements for fire safety are related to the number of resident beds in each sub compartment, providing a ratio of approximately one staff member for each two beds in a sub compartment up to a maximum of nine beds |
| Schnitker et al. (2017)  A national survey of aged care facility managers’ views of  preparedness for natural disasters relevant to residents with dementia  Online survey (n=416) | Personal factors   - Managers felt strongly that residents with dementia had special needs during a natural disaster or evacuation. (b) - Managers felt that this population was more vulnerable due to a potential for increased confusion, expressed as a lack of insight into the nature of the emergency or an inability to understand or follow instructions, and/or for behavioural symptoms, such as anxiety, distress, wandering or agitation. (b) - Managers were concerned that these cognitive and behavioural issues could adversely impact other residents, staff and the overall effectiveness of the disaster management or evacuation. (b)   Environment factors   - Not discussed   Occupation – evacuation practices:   - Identification of a gap between perceived preparedness as documented in disaster plans and real preparedness as demonstrated by necessary training and drilling and dementia-specific awareness. - Two-thirds of the participants indicated that their staff had been trained in disaster management in the previous 12 months. These exercises included primarily practical fire training and emergency management. - 82% of managers specified that their facility had conducted evacuation practice drills (mock evacuations) during the last 12 months. In those drills, residents were involved in 45% of the cases. - For people with dementia, they recommended:   - Calm and proper instructions, constant reassurance, and close supervision   - Clear identification for each resident   - To include a specific reference in the management plan for dealing with communication, identification, and behavioural symptoms. |
| Turner et al. (1991) Preparation for a Fire Disaster in a Long-term Care facility: A staff Development Perspective.  Case study | Personal factors   - People on intravenous drugs or respiratory aids will be very difficult to evacuate, especially if the weather is not good (b)   Environment factors   - Poorly organised evacuation manuals make it impossible to find information (b) - Evacuation maps on poorly lit walls or not up to date (b) - No card ready to indicate that the room is empty (b) - Locked doors without keys easily available (b)   Occupation – evacuation practice:   - A lot of simulation are done with attendants only, no practices with seniors (lot of stress), But this one shows that good preparation can reduce the risk of problems for residents, highlight problematic factors and errors and give confidence to attendants. Practicing an evacuation is essential to help learn how to do it right. |

Table 5: Results extracted from grey literature (date) according to barriers, facilitators and current evacuation practices

| **Authors (year)**  **Title**  **Type of document** | **Barriers (b), Facilitators (f) and Current evacuation practice** |
| --- | --- |
| Carrara (2010)  Community-Based Nursing Plan to Help Seniors Safely Evacuate from an Independent Living Facility in an Emergency  Doctoral dissertation | Personal factors   - Persons with mobility impairment may be at high risk of falls as they attempt to descend stairs. While elevators are the safest route of evacuation for those with mobility impairments, elevators are inoperable and inaccessible during a fire or other disaster (b) - A barrier to mobility and evacuation is the inability to digitally manipulate locks, latches, and/or handles that are found on many emergencies escape routes. Arthritis is an inflammatory condition of the joints that can affect the ability to use the hands and the ability to grasp. (b) - Seniors with respiratory dysfunction may have trouble with evacuation. Respiratory dysfunction impairs oxygen exchange, therefore those who suffer from respiratory illness and dysfunction can quickly become short of breath. A shortage of oxygen causes symptoms such as dizziness, inability to concentrate, and fatigue, all of which are barriers to a safe evacuation. When fire and smoke are present, symptoms of respiratory dysfunction and distress are exacerbated. (b) - Visual disturbances would affect one's ability to see and read signs that are pertinent in understanding where to go and what to do during an evacuation. Furthermore, being in a dark building or having to evacuate during the night poses additional challenges. (b) - For those with an auditory impairment, there is a question about whether or not they would hear a sounding alarm during an emergency. Those with hearing loss may miss pertinent information regarding the situation or fail to hear vital instructions that assist with safe evacuation. (b) - Cognitive deficits can contribute heavily to the lack of safety of older adults during evacuation. These deficits can affect one’s ability to remember instructions or plan, cause difficulty responding to events in an immediate timeframe, and affect their response to stimuli. (b) - A person with Alzheimer’s disease may become disoriented, may not understand the significance of the alarm sounding, or may misinterpret the sound of the alarm and its significance. In this situation, the individual may lose his or her ability to judge environmental cues or safely (b) - Sleep inertia can be defined as the decrease or impairment of performance that occurs immediately upon awakening from sleep compared to that prior to sleep. It reduces decision-making performance for at least 30 minutes and therefore may affect the evacuation (b) - Medications can play an enormous part of whether or not a community member would be able to safely evacuate and respond to emergencies. Older adults may be taking a large quantity of medications on a daily basis that may interfere with reaction time and alertness. (b)   Environment factors   - Some alarm systems will include a strobe light for those that are hearing impaired, however there is no guarantee that the occupant would know what action to take upon recognition of the alarm without written instructions (f) - In anticipation of an emergency, a chain of command should be in place to facilitate decision-making; to do otherwise may lead to chaos (f) - One of the most important evacuation components is that of a specific plan or procedure. Making sure that all of the residents are familiar with escape routes is essential (f) - The escape route needs to be clearly marked with appropriate signage. These signs should include large font that is colored to stand out against its background. The language should be clear and concise in order to be easily understood by residents. Including reflective material on signage enables individuals to better discern the written message under dark conditions or when there is only a scant amount of light. (f) - Handrails must be in place to assist those with mobility impairments. (f) - Detailed mapped escape routes should be posted in plain sight throughout the buildings, such as just outside of the escape route entrance, stairway entrances, and other egress routes (f)   Occupation – evacuation practices:   - The U.S. Fire Administration (2001) has identified that 1,200 older adults die annually from fire; fire is the 6th leading cause of death among older adults. Finally, 85 percent of fire deaths in 2009 occurred in homes and cooking was the primary cause (Karter, 2010). - Community health nurses are in prime position to help craft plans that reduce the vulnerability of older adults during an emergency; effective planning and patient education can reduce the risk of injury and death due to disasters such as hurricanes, building fires, and earthquakes. - Merely providing written or verbal instruction upon establishment of residency into an independent living facility may be an insufficient means of ensuring evacuation safety. Therefore, it is essential to practice emergency drills in order to build familiarity especially for those with significant limitations. - Incorporating as many senses as possible in the education will ensure that everyone will have an opportunity to learn the way that they learn the best. For instance, some individuals learn better by doing, while others may learn better by seeing, hearing, writing, or verbalizing When providing written material, it is helpful to use a larger font that greatly contrasts with the background colors of the page. Fonts that are basic in nature rather than embellished are preferred. Perry and Potter (2009) suggest incorporating audio, visual, and tactile methods of learning to increase |
| Direction de la sécurité incendie et des télécommunications d’urgence et du Service du développement en sécurité incendie du ministère de la santé public du Québec (2021)  Statistiques à propos des incendies au Québec  Summary of government data | Personal factors   - Not discussed   Environment factors   - The vast majority of fires started in residential areas (except storage areas and garages) followed by service or commercial areas - The vast majority of residential fires in RPAs started in the kitchen, followed by the bedroom - The main source of heat causing fires in RPAs is cooking appliances, followed by electrical appliances, and smoking or open-flame items - The most likely cause of fire in RPAs is human error, followed by electrical failure or malfunction - In the majority of fires in RPAs between 2016-2019, smoke alarms worked - In the majority of fires in RPAs between 2016-2019, the alarm system worked   Occupation – evacuation practices:   - Not discussed |
| ​​Ducharme (2005)  Du logement à l’hébergement : les régulations dans l’aménagement, les immeubles et les services  Final research report (FQRSC) | Personal factors   - Not discussed   Environment factors   - Facilities such as evacuation protocols evacuation protocols, heat and smoke detectors and emergency exit (MAMM, 2003) are mandatory under the National Fire Code. (f) - The measures may consist of security systems for access to buildings and dwellings (e.g., intercoms, locked doors, cameras) or fire and evacuation safety measures in case of fire or evacuation (heat and smoke detectors, fire extinguishers, fire extinguishers, emergency exits, sprinklers, evacuation plan) and on-call systems for people (pull cord) for people (alarm pulls). (f) - Based on observations in the homes studied, most have sprinklers. Security systems at the entrance may take the form of reception counters, locked doors with intercom systems or surveillance cameras. Low-income housing seems to opt more for locked entrance door systems, while private residences are equipped with reception counters or surveillance cameras. As for emergency call systems linked to a central station, they are more common in private for-profit residences (f) - Although the security systems are not so elaborate in private residences, the residents all feel safe there since the absence of systems is compensated for by the continuous presence of staff members. (f) - On the other hand, residents of low-income housing located in urban areas (southwest of Montreal and Longueil) complained about the lack of security, particularly for access to the building. (b)   Occupation – evacuation practices:   - The mandatory measures are observed in all the residences under study. It is the responsibility of the municipalities to ensure their inspection. The other measures and their application are more are more random. |
| Gilmartin et al. (2019)  Improving disaster preparedness, response, and recovery for older adults.  evidence-informed recommendations summary | Personal factors   - Not discussed   Environment factors   - Not discussed   Occupation – evacuation practices:   - This article gives recommendations on 5 different aspects in relation to evacuation and prevention: - Individuals and Caregiver: 9 recommendations - Health Care Professionals and Emergency Response Personnel: 2 recommendations - Community Based Services and Programs: 3 recommendations - Care Institutions and Organizations: 2 recommendations - Legislation/Policy Domain: 6 recommendations - Research: 3 recommendations |
| Gouvernement du Québec (2008)  Guide pratique: la prévention des incendies et l’évacuation des résidences hébergeant des personnes âgées  Government guide | Personal factors   - 1st stage (detection) limitation (b):   - Not hearing the alarm (hearing problems, sleeping medication, isolated room or low alarm volume)   - Not understanding (lack of information/training, cognitive problems)   - Not being able to call for help due to lack of communication system   - Not starting the evacuation immediately (too many false alarms, alarm interrupted prematurely...)   - Not knowing what to do (lack of information/training) - 2^nd^ and 3^rd^ stage (understanding and moving) limitation (b):   - Not being able to leave bed (physical disability, contention)   - Not being able to leave the home and evacuate horizontally or vertically (physical disabilities)   - Not being able to move in corridors or stairs (physical disabilities)   Environment factors   - The impossibility of a horizontal evacuation (b) - Difficulties in detecting fire (b) - Lack of a linked alarm system (b) - Narrowness of exits (b) - Lack of sprinklers (b) - Not being able to leave the home and evacuate horizontally or vertically (clutter, crowding, closed/locked doors, lack of handrails, lack of visibility) (b)   Occupation – evacuation practices:   - The 4 stages of an evacuation: - The detection of the fire and the raising of the alarm - Understanding and deciding to undertake the evacuation - Moving - Relocation - Staff training and information to residents   - Once the fire safety plan has been completed, it may be necessary to review the fire safety training for staff, particularly fire safety procedures in the event of a disaster. It will also be important to teach these procedures to residents. - The regulations stipulate that evacuation instructions for residents must be posted on each floor of the residence in a residence, in a place accessible to the public. It is suggested that they be placed near a representation of the evacuation routes. |
| Gouvernement du Québec (2017)  Sécurité incendie dans les résidences accueillant des personnes présentant des limitations à l’évacuation  Government guide | Personal factors   - I ensure that my employees and replacements have received fire safety training (f) - I inform my residents about fire safety and good behaviour (f)   Environment factors   - Not discussed   Occupation – evacuation practices:   - I establish my evacuation strategies (f) - I carry out fire drills every 6 months (f) |
| OPHQ (2017)  L'habitation Évaluation de l'efficacité de la politique gouvernementale: À part entière: pour un véritable exercice du droit à l'égalité  Government guide | Personal factors   - Not discussed   Environment factors   - There is an inadequacy and inappropriateness of safety measures appropriate to the varied and changing circumstances of people with disabilities, regardless of where they live or are housed. Indeed, the Regulation to improve safety in the building provides for more restrictive fire safety requirements than the initial requirements for independent RPPs subject to the MSSS certification process. Owners of RPPs for non-autonomous clients must comply with stricter requirements for care facilities (hospitals, Long-term Care Home) or supervised residences (ex: mandatory sprinkler installation). (b)   Occupation – evacuation practices:   - Not discussed |
| Service de la sécurité incendie (2001)  Complément au guide pratique: La prévention des incendies et l’évacuation des résidences hébergeant des personnes âgées, informations destinées aux exploitants.  Government guide | Personal factors   - Not discussed   Environment factors   - Combustible construction (b) vs Non-combustible construction (f) - Ways to limit the spread of fires (f): Sprinkler system, fire separation and fire wall - Types of detection and alarm equipment (f): Smoke alarm, fire detection, alarm system, smoke detectors (single signal and dual signal ) and carbon monoxide alarm or detector - Other equipment strongly recommended (f): - Emergency lighting in exits, corridors, and stairwells - Choose furniture and textile materials with a low flammability and smoke development index. - The means of communication (f): voice communication network and communication device between employees - For combustible construction, the safety places are mainly located outside the building.   Occupation – evacuation practices:   - The purpose of a fire safety plan is to promote fire prevention and, through the measures it contains, the efficient, orderly and safe evacuation of residents to safe locations during a fire:   - Improve knowledge of the roles and responsibilities of the various players involved in the exercise and the use of equipment to facilitate the evacuation.   - Promote the rapid response of the IMS, employees, and agencies through the automated transmission of any alarms.   - Coordinate the action and exchange of information among all stakeholders.   - To consolidate the collaboration links with the staff, the residents, and the partners. - The proposed evacuation times consider, among other things, the type of construction and characteristics of the building and the fire alarm and safety equipment. These times are 3 (buildings with minimal protection), 5, 8 or 11(presence of a complete and efficient fire detection and alarm system) minutes. |
| Somes et al. (2012)  Disaster Planning Considerations Involving the Geriatric Patient: Part 1  Review of the situation of elderly people in case of fire | Personal factors   - Many of the patients had dementia, several were wheelchair bound, and all had some sort of underlying medical condition or required help with activities of daily living (b) - Older adults may not be able to hear, process instructions they are given, or even remember what they were told to do. It is particularly true if the patient has dementia or short-term memory problems. Older adults may not be able to see, read, or follow instructions on signs. They may not be able to move to a safe place on their own. (b) - High anxiety conditions make memory issues worse. (b)   Environment factors   - Not discussed   Occupation – evacuation practices:   - The noise and confusion of a rapid evacuation can lead to the older adult moving even more slowly, carefully, and deliberately. - Rapid evacuation to safety leads to increased risk of falls, or the “less-than-gentle moving” of the older adult. This puts the older adult’s fragile skin, joints, and bones at risk of injury. Matching evacuation speed to a level that makes it comfortable for the seniors puts all involved at greater risk of prolonged exposure to danger |
| Zork (2014)  Nursing Home Disaster Planning and Response: A Policy Perspective  policy perspective | Personal factors   - Evacuation and transfer results in increased stress to cognitively impaired nursing home residents in part because it is more difficult to prepare these residents for the event. (b)   Environment factors   - Not discussed   Occupation – evacuation practices:   - Nursing home emergency management is an ongoing and dynamic process that comprises four phases:   - Mitigation activities involve the reduction of exposure during high-risk events. Some examples include zoning and building code requirements in high-hazard areas.   - Preparedness activities equip facilities to respond when threats occur by establishing responsibilities for emergency actions and allocating the resources to support them. Preparedness also requires conducting meaningful drills and exercises to educate personnel, as well as testing plans and equipment.   - Response activities reduce the negative impact to residents and staff when emergencies occur and include communicating with emergency management personnel, evacuating or sheltering, and caring for residents as routinely as possible during the emergency.   - Recovery is the effort to restore resident life to normal following a disaster. - Plan may be reviewed and revised as often as needed, but at least annually. - Management of patients with dementia may include using private rooms for distressed residents, having social workers on staff to provide support, surrounding residents with familiar individuals when possible, using appropriate lighting, and providing activities to residents with dementia during evacuation |

Table 6: Resulted extracted form patents found in the grey literature review (date)

| **Authors (Year) Patent name (patent number)** | **Description of the equipment or patent** |
| --- | --- |
| BROOKS (1987)  Evacuation chair ([US4711487A](https://worldwide.espacenet.com/patent/search?q=pn%3DUS4711487A)) | - The invention enables the transporting of a handicapped person by two or more other persons more easily, more securely and with less discomfort to the handicapped person than is the case where manual carrying must be performed without the aid of equipment for facilitating the task. While not being limited to such usage, the apparatus is particularly adaptable to the carrying of handicapped persons down stairways during a building evacuation. The contractability of the apparatus makes it practical to stock high rise buildings or the like with the devices which may, if desired, be stored in conspicuous locations with identifying instructions which make the purpose of the device and its operation readily apparent to persons who may not be trained in evacuation techniques and procedures. |
| Cameron (1994)  An evacuation means ([AU5046393A](https://worldwide.espacenet.com/patent/search?q=pn%3DAU5046393A)) | - This invention relates to an evacuation means, and, in particular, an evacuation means which incorporates a mattress or combines with a bed mattress in a particularly useful manner to create a means for transport of patients from hospitals and the like.   The invention is advantageous in the form of an evacuation means which can combine with a mattress in a particularly useful manner to create a patient handling apparatus which cocoons the patient in a padded stretcher-like manner. |
| [Howitt](https://patents.google.com/?inventor=Kevin+Howitt) (2004)  Emergency Evacuation Apparatus for a Bed-Ridden Person (US20080115283A1) | - The invention relates to an improved evacuation apparatus to enable the rapid and safe removal of bedridden people from hospitals, old persons homes and other buildings in emergency situations. - The invention provides an emergency evacuation apparatus for removing a bed-ridden person from a bed in an emergency. The apparatus includes a base tray having at least one transverse fold line to allow the base tray to bend as two or more panels. This apparatus is therefore suitable for use with a modern adjustable bed, which can be electrically or manually operated to adjust the profile of the mattress to provide better ergonomic support for the person lying on the bed. The base tray also has mechanical locking means operable between an unlocked condition where the base tray can bend along the transverse fold line and a locked condition where the base tray is unable to bend along the transverse fold line. The mechanical locking means also provides the base tray with sufficient longitudinal rigidity to support the weight of an adult person. |
| Kenalty et al. (2013)   Evacuation Sled ([US2013276227A1](https://worldwide.espacenet.com/patent/search?q=pn%3DUS2013276227A1)) | - An evacuating transfer device includes: a rail annularly mounted on an upper part of a staircase of a building, from the lower floor to the upper floor; a plurality of carriages travelling on the rail, which include a suspension member for suspending a person or a weight body being a transfer object via a suspension tool and to which a linking member can be attached for linking the plurality of carriages |
| Meng (2014)  Stair-climbing chair equipped with power and convenient to make way ([CN203877695U](https://worldwide.espacenet.com/patent/search?q=pn%3DCN203877695U)) | - The utility model provides a new device called "the chair with self-contained power and convenient avoidance", which is a major improvement. It is characterized by solving the problem of continuous building and avoidance of multi-storey houses: the self-powered boarding chair can self-go from the starting point of the bottom floor track, continuously climb the top floor track terminal along the track, or from the top floor to the bottom floor track terminal. |
| Olson et al. (2013)  Apparatus and Method for the Transfer and Movement of Objects ([US2013227789A1](https://worldwide.espacenet.com/patent/search?q=pn%3DUS2013227789A1)) | - The present invention relates to a method and apparatus for transporting people, animals and objects, including people and animals with disabilities or who have difficulty in moving themselves. More specifically, the present invention typically utilizes a sling-type device in a variety of configurations for moving people, animals, and other objects. - By ensuring more ergonomic lifting positions and better weight distribution, the present invention potentially reduces the likelihood of back injuries and other related injuries to firefighters, Emergency Medical Services provider (“EMS”), and others who are involved in moving patients and other heavy objects. |
| Park (2012)  Emergency evacuation device for building ([KR101195437B1](https://worldwide.espacenet.com/patent/search?q=pn%3DKR101195437B1)) | - The present invention relates to an emergency escape apparatus for a building, and more particularly, to an escape apparatus in an emergency staircase or a designated evacuation area so that a person in a building in an emergency, such as a fire in a building, The emergency escape apparatus can be folded or folded so that the escape apparatus can be folded or folded so as to reduce the volume of the escape apparatus after the escape personnel gets off. To an emergency escape apparatus capable of easily evacuating the emergency escape apparatus. |
| Seo (2014)  Emergency evacuation device  [KR20140107877A](https://worldwide.espacenet.com/patent/search?q=pn%3DKR20140107877A) | - The present invention relates to an emergency escape device, and more particularly, an emergency escape device that can move between floors in an emergency such as a fire, so as to safely escape from the general public, pregnant women, children, patients with inferior limbs or the elderly, etc. It is about an escape device. |
| Shibukawa (2003)  Carrying device and care facility  ([JP2003088560A](https://worldwide.espacenet.com/patent/search?q=pn%3DJP2003088560A)) | - The present invention relates to a transport device and a nursing care facility. That can be easily transported or transported to the outside for each wheelchair or stretcher. - According to a first aspect of the present invention, there is provided a transport apparatus for connecting a plurality of traveling lift means to a plurality of traveling lift means at a predetermined interval. And a connecting means for integrally moving along a trajectory. According to a second aspect of the present invention, there is provided the transporting apparatus according to the first aspect, wherein the traveling lift means suspends a wheelchair on which the care receiver rides. According to a third aspect of the present invention, there is provided the transport apparatus according to the first aspect, wherein the traveling lift means is a set of two units and suspends a stretcher on which the care receiver is placed. Things. According to a fourth aspect of the present invention, there is provided the transporting apparatus according to any one of the first to third aspects, wherein the traveling lift means is an electric hoist. |
| Spiller (2007)  Apparatus for use in an evacuation chute ([GB2451129A](https://worldwide.espacenet.com/patent/search?q=pn%3DGB2451129A)) | - It is important to appreciate that even if the patient had had a spinal injury or had been immobilized for any other reason, the above-described evacuation procedure would have been feasible, even to the point of moving the apparatus down stairs or through a window, because the base tray provides all the rigidity necessary to keep the patient in an immobilized condition during evacuation. - The invention relates to an improved evacuation apparatus to enable the rapid and safe removal of bedridden people from hospitals, old persons homes and other buildings in emergency situations. |
| Walkingshaw et al. (2008)  Evacuation Sled and Temporary Surge ([US2008265557A1](https://worldwide.espacenet.com/patent/search?q=pn%3DUS2008265557A1)) | - A lightweight, strong, sturdy, and versatile evacuation sled is described. The surfaces of the sled may be manufactured from a single piece of material, stored flat, and then folded into the sled's functioning configuration. When folded, the sled has one or more straps that help hold the sled in its configuration and provide points for attachment to the sled and for maneuvering or carrying the sled. The sled may be readily towed with a towing harness and may be moved down an incline, such as stairs, by means of a rope and belay device. The sled has a low coefficient of friction and is lightweight. This allows the sled to be readily moved and handled without requiring the assistance of multiple people. |
| Napanju (2011)  Emergency escape device (WO2011122817A2) | - The present invention relates generally to devices for existing structures during emergency situations and, more particularly, to a novel system for assisting otherwise trapped persons in a high-rise structure. - A system for the evacuation of persons from a multi-story structure during circumstances in which the normal escape mechanisms such as stairs and elevators are unusable for any reason. The system mounts externally of the affected structure and provides at least one evacuator upon which a person may ride in a controlled descent from an area of danger to an area of relative safety. The evacuator rides on a post and the evacuee may control the speed of his or her descent with a simple braking mechanism. A safety belt and footrests provide a sense of security during the descent from danger. |
